# The association between abcb1 gene polymorphism and clopidogrel response variability in stroke ischemic: a cross sectional study

**DOI:** 10.1186/s12883-024-03723-y

**Published:** 2024-06-24

**Authors:** Rakhmad Hidayat, Rizqi Amanda Nabilah, Marc Fisher, Tiara Aninditha, Mohammad Kurniawan, Riwanti Estiasari, Luh Ari Indrawati, Ahmad Yanuar Safri, Taufik Mesiano, Al Rasyid, Salim Harris

**Affiliations:** 1 Department of Neurology, Faculty of Medicine, Universitas Indonesia, Jakarta, Indonesia; 2 Dr. Cipto Mangunkusumo Hospital, Jakarta, Indonesia; 3 Harvard Medical School, Boston, USA

**Keywords:** ABCB1, Clopidogrel, Response variability

## Abstract

**Background:**

Clopidogrel has been the primary choice of antiplatelet in ischemic stroke that inhibits *adenosine diphosphate* (ADP)-induced platelet aggregation. P-glycoprotein (P-gp) *multidrug resistance-*1 (MDR1) is a transmembrane efflux transporter in intestinal cells that plays a significant role in clopidogrel absorption, therefore may affect platelet aggregation. P-gp is encoded by the ABCB1 gene. This study aims to evaluate the effect of ABCB1 polymorphism on clopidogrel response variability in ischemic stroke patients and its genotype frequency.

**Methods:**

A cross-sectional study was conducted in ischemic stroke patients who received clopidogrel between 2020 and 2023 in RSUI/RSCM. All subjects were assessed for ABCB1 polymorphisms C3435T and C1236T. Platelet aggregation were measured using VerifyNow PRU. Clopidogrel response variability was classified into unresponsive (> 208 PRU), responsive (95–208 PRU), and bleeding risk (< 95 PRU).

**Results:**

124 subjects enrolled in this study, with 12,9% of subjects classified as non-responsive/resistant, 49,5% as responsive, and 41,9% as bleeding risk. ABCB1 C1236T homozygote wildtype (CC) was associated with 3,76 times higher bleeding risk than other variants (*p* = 0,008; 95%CI 1,41 − 10,07). Genotype frequency of ABCB1 C3435T homozygote wildtype, heterozygote, and homozygote variants were 35,9%, 43,5% and 16,9%, respectively; while the genotype frequency of ABCB1 C1236T were 17,8%, 39,5%, and 42,7%, respectively.

**Conclusion:**

ABCB1 C1236T homozygote wildtype was associated with 3,76 times higher bleeding risk than other variants. The most common genotype frequency of ABCB1 C1236T was homozygote variant; while for ABCB1 C3435T was heterozygote.

## Introduction

Ischemic stroke remains one of the most common causes of disability and mortality in Indonesia and worldwide. Antiplatelet therapy remains the primary choice for recurrent stroke prevention, with the American Heart Association/American Stroke Association (AHA/ASA) recommending the use of aspirin and/or clopidogrel for atherosclerotic stroke [1–5]. Paciaroni et al. found that single antiplatelet therapy using clopidogrel is superior than aspirin in terms of reducing the recurrence of cardio-cerebrovascular disease and bleeding risk [6]. Nonetheless, the effectiveness of clopidogrel is occasionally hindered by resistance, resulting in recurrent strokes.

Clopidogrel inhibits platelet aggregation induced by adenosine diphosphate (ADP) by acting as a blocker of the P2Y12-receptor. This inhibition subsequently disrupts platelet activation, degranulation, and aggregation by inhibiting the glycoprotein IIb/IIIa receptor [7]. Clopidogrel is ingested as a prodrug then metabolized in the liver before reached its active form. Many factors subsequently influence the effectiveness of clopidogrel, including absorption, activation, and metabolism. These factors are divided into cellular, genetic, and clinical factors. The combined influence of these factors leads to varied individual responses to clopidogrel treatment, known as clopidogrel response variability, which can result in clopidogrel resistance and increase the risk of cardiovascular complications or even bleeding [8, 9].

Clopidogrel mainly works in three primary locations: the intestines, liver, and platelets. Clopidogrel is absorbed in the intestines under the regulation of P-glycoprotein (P-gp), a product of the ATP-binding cassette subfamily B member-1 (ABCB1) gene. Subsequently, clopidogrel is activated by the liver through various cytochrome enzymes (CYP450) in two steps. In the first step, clopidogrel is converted into 2-oxo-clopidogrel, which is an inactive intermediate metabolite, by CYP1A2 (35.8%), CYP2B6 (19.4%), and CYP2C19 (44.9%). In the second step, 2-oxo-clopidogrel is transformed into R-130,964 (clop-AM) by CYP2B6 (32.9%), CYP2C9 (6.79%), CYP2C19 (20.6%), and CYP3A4 (39.8%), which is the active metabolite form of clopidogrel [10, 11]. The active metabolite of clopidogrel then binds irreversibly to the P2Y12 receptor, leading to the inactivation of GP IIb/IIIa and reducing thrombus stability, thereby prevents platelet activation [9].

The role of genetic polymorphism begins with its impact on clopidogrel absorption in the body. The ABCB1 gene is part of the ATP-binding cassette (ABC) transporter group, encoding the multidrug resistance-1 (MDR1) P-glycoprotein transporter. P-gp or MDR1 is a transmembrane protein whose primary function is to pump drugs out of cells into the bloodstream. Polymorphisms in ABCB1 can affect P-gp levels, leading to reduced clopidogrel absorption, decreased drug activity, and an increased risk of cerebrovascular events and death [8, 12–17]. Several single nucleotide polymorphisms (SNPs) have been identified and are said to be associated with clopidogrel resistance, including C1236T, G2677T/A, and C3435T. Among these three SNPs, C3435T is reported to be most commonly linked to clopidogrel resistance. This polymorphism does not induce changes in amino acids but rather alters splice sites, subsequently affecting mRNA protein formation [15–17].

High on-treatment platelet reactivity (HPR), or increased platelet reactivity, is a phenomenon that can occur as a result of clopidogrel resistance. HPR is significantly associated with the risk of thrombosis and mortality. The prevalence of clopidogrel resistance varies, with Asian populations having the highest rates compared to Caucasians and African Americans. Studies in Indonesia have found a prevalence of clopidogrel resistance ranging from 15.8 to 33% in ischemic stroke patients. Differences in prevalence can be attributed to demographic factors, genetic polymorphisms, drug interactions, and comorbidities that influence the pharmacokinetics and pharmacodynamics of clopidogrel [9, 19–21].

This study aims to investigate the relationship between ABCB1 gene polymorphisms and variability in clopidogrel response among ischemic stroke patients, particularly related to its role in clopidogrel absorption. The investigation into ABCB1 in the context of ischemic stroke is likely focused on understanding how this transporter protein may impact the efficacy of drugs used in stroke treatment. Response variability in ischemic stroke patients can be influenced by factors such as genetic variations in drug-metabolizing enzymes and drug transporters, including ABCB1 The study will also assess the frequency of ABCB1 gene polymorphisms, particularly single nucleotide polymorphisms (SNPs) C1236T and C3435T.

By understanding the genetic factors that influence drug response, researchers and clinicians can tailor treatment strategies based on an individual’s genetic makeup, ultimately aiming for more personalized and effective therapies. This approach, known as pharmacogenomics, seeks to optimize drug selection and dosage to improve treatment outcomes while minimizing adverse effects.

Clopidogrel is a thienopyridine class antiplatelet that acts by inhibiting the binding of ADP to the P2Y12 receptor. This inhibition subsequently disrupts platelet activation, degranulation, and aggregation by inhibiting the glycoprotein IIb/IIIa receptor [7].

The role of genetic polymorphism begins with its impact on clopidogrel absorption in the body. The ABCB1 gene is part of the ATP-binding cassette (ABC) transporter group, encoding the multidrug resistance-1 (MDR1) P-glycoprotein transporter. P-gp or MDR1 is a transmembrane protein whose primary function is to pump drugs out of cells into the bloodstream. Polymorphisms in ABCB1 can influence P-gp levels, causing a decrease in clopidogrel absorption, resulting in variability in treatment response [15–17].

Several single nucleotide polymorphisms (SNPs) have been identified and are said to be associated with clopidogrel resistance, including C1236T, G2677T/A, and C3435T. Among these three SNPs, C3435T is reported to be most commonly linked to clopidogrel resistance. This polymorphism does not induce changes in amino acids but rather alters splice sites, subsequently affecting mRNA protein formation.^15–17^

## Methods

This study is a developmental study titled “Performance Scoring of Clopidogrel Effectiveness in Assessing Platelet Aggregation with Modified STIB Score in Ischemic Stroke Patients Linked to Cytochrome P450 Concentration and CYP2C19 Polymorphism, as well as P2Y12 Receptor [18]. It employs a cross-sectional design using primary data from the initial study. Research was conducted on ischemic stroke patients who received clopidogrel therapy and were treated at dr. Cipto Mangunkusumo National General Hospital (RSUPN dr. Cipto Mangunkusumo) or the University of Indonesia Hospital (RS Universitas Indonesia) from 2020 to 2023.

The inclusion criteria were as follow patients aged > 18 years old who had taken clopidogrel for a minimum of 5 days and agreed to participate in the study. Exclusion criteria included the use of thienopyridine antiplatelet drugs other than clopidogrel, platelet counts < 150,000 or > 450,000/uL, and patients currently undergoing chemotherapy. Samples were stored in the form of buffy coats, and DNA extraction was performed. Subsequently, eligible samples were examined for ABCB1 gene polymorphisms C3435T and C1236T. All samples (*n* = 124) were stored in a freezer at -80 °C and were suitable for analysis. All patients underwent clopidogrel response testing with VerifyNow PRU, as well as the analysis of ABCB1 gene polymorphisms C1236T and C3435T using PCR. Response variability will be assessed based on platelet aggregation function using VerifyNow, which then classified as resistant, non-resistant, or bleeding risk. VerifyNow PRU testing is a rapid and reliable method for assessing platelet response to clopidogrel, an antiplatelet medication used to prevent blood clot formation in cardiovascular patients. By measuring platelet reactivity and inhibition of the P2Y12 receptor, VerifyNow PRU provides clinicians with valuable information to guide treatment decisions, such as adjusting dosage or selecting alternative therapies.

Statistical analysis was carried out using IBM Statistical Package for Social Science/SPSS Statistics version 20. Numeric data were analyzed using independent T-tests or ANOVA if normally distributed, and Mann-Whitney or Kruskal-Wallis tests if the distribution was not normal. Categorical data were analyzed using Chi-square or Fisher’s exact tests. The confidence level used was 95%, or *p* = 0.05. This research has received ethical approval (registration number: KET-658 from the Research Ethics Committee of the Faculty of Medicine, University of Indonesia - RSUPN Dr. Cipto Mangunkusumo.

## Results

A total of 128 subjects with ischemic stroke who were taking clopidogrel at Cipto Mangunkusumo Hospital (RSCM) and the University of Indonesia Hospital (RSUI) were enrolled in the initial study. All patients underwent medical history assessment, physical examinations, diagnostic tests, clopidogrel resistance testing, and examinations for P2Y12 receptor resistance and CYP2C19 gene polymorphism.

Four subjects were excluded due to incomplete data, resulting in a final sample size of 124 subjects, with 28 subjects from RSCM and 96 subjects from RSUI.

### Baseline characteristics

Subjects were divided into three groups based on their variability in response to clopidogrel (Table 1), which was assessed using VerifyNow: non-responsive (> 208 PRU), responsive (95–208 PRU), and bleeding risk (< 95 PRU). A total of 12.9% of the subjects were categorized as non-responsive to clopidogrel, 45.2% as responsive to clopidogrel, and 41.9% as having bleeding risk with clopidogrel administration. There was no significant difference of mean age among the three groups.

Based on the baseline clinical characteristics (Table 1), the subjects’ characteristics were similar across all three groups for almost all variables, except for dyslipidemia. A significant difference in the proportion of dyslipidemia was observed among the groups, with the highest proportion in the non-responsive or clopidogrel-resistant group (*p* = 0.044).

**Table 1:** Clinical Characteristics of Subjects with Clopidogrel Responsiveness Variability (*n* = 124)

Parameter	Mean (+- SD) Non-responsive *N* = 16	Responsive *N* = 56	Bleeding risk *N* = 52	*p*
Age	59.85±7.63	59.78±11.49	57.98±10.28	0.281^a^
Gender-N (%)				0.140^b^
Male	6 (37.5)	33 (58.9)	34 (65.4)	
Female	10 (62.5)	23 (41.1)	18 (34.6)	
Smoking	11 (68.8)	22 (39.3)	26 (50)	0.324^b^
Yes	5 (31.3)	34 (60.7)	25 (50)	
No				
History of aspirin use	5 (31.3)	27 (48.2)	24 (46.2)	0.477^b^
Yes	11 (68.8)	29 (51.8)	28 (53.8)	
No				
History of cilostazol use	1 (6.3)	1 (1.8)	6 (11.5)	0.121^c^
Yes	15 (93.8)	55 (98.2)	46 (88.5)	
No				
History of stroke	6 (37.5)	15 (26.8)	15 (28.8)	0.706^b^
Yes	10 (62.5)	41 (73.2)	37 (71.2)	
No				
Type 2 DM	6 (37.5)	24 (42.9)	17 (32.7)	0.553^b^
Yes	10 (62.5)	32 (57.1)	35 (67.3)	
No				
Hypertension	10 (62.5)	38 (67.9)	31 (59.6)	0.669^b^
Yes	6 (37.5)	18 (32.1)	21 (40.4)	
No				
Dyslipidemia				**0.044** ^**b**^
Yes	13 (81.3)	26 (46.4)	30 (57.7)	
No	3 (18.8)	30 (53.6)	22 (42.3)	
Dyspepsia				0.461^b^
Yes	3 (18.8)	16 (28.6)	18 (34.6)	
No	13 (81.3)	40 (71.4)	34 (65.4)	
BMI				0.214^c^
< 18.5	2 (14.3)	2 (3.6)	2 (3.9)	
18.5–25	9 (64.3)	28 (50.9)	25 (49)	
25.1–27	0 (0)	6 (10.9)	11 (21.6)	
> 27	3 (21.4)	19 (34.5)	13 (25.5)	
Fatty liver				0.471^b^
Yes	10 (62.5)	27 (48.2)	30 (57.7)	
No	6 (37.5)	29 (51.8)	22 (42.3)	
Uric acid				0.182^b^
Yes	1 (6.2)	16 (28.6)	13 (25)	
No	15 (93.8)	40 (71.4)	39 (75)	

Data is presented as n(%) for categorical variables and mean ± standard deviation for numeric variables; *p* < 0.05. ^a^One-way ANOVA; ^b^Chi-square; ^c^Kruskal-Wallis

Based on laboratory examinations (Table 2, a significant difference was found in hemoglobin levels in relation to clopidogrel response variability (*p* < 0.001). with the highest mean value observed in the bleeding risk group. The median VerifyNow value was 243 (216–272) in the non-responsive group. 134 (97–195) in the responsive group, and 56.5 (1–93) in the bleeding risk group. No significant differences were observed in total cholesterol. high-density lipoprotein (HDL), low-density lipoprotein (LDL), body mass index (BMI), fasting blood sugar, HbA1c, triglycerides, ALT, AST, urea, and creatinine.

**Table 2:** Laboratory Parameters and Clopidogrel Response Variability (*n* = 124)

Parameter	Non-responsive (Mean±SD)	Response (Mean±SD)	Bleeding risk (Mean±SD)	*p*
Haemoglobin	12.74±1.08	13.92±1.31	14.27±1.74	**< 0.001** ^**a**^
LDL cholesterol	205.62±42.29	217.73±259.87	186.07±51.55	0.491^c^
Total cholesterol	211 (138–277)	182 (93-2020)	200 (100–312)	0.224^c^
HDL cholesterol	59 (32–102)	50.5 (26–70)	49.5 (22–85)	0.754^c^
BMI	23.4 (18-33.1)	24.45 (16.6–45.8)	24.65 (15.6–31.2)	0.490^c^
Fasting glucose	94.5 (80–280)	118.7 (72.9–331)	106.5 (77–449)	0.089^c^
HbA1c	5.5 (4.6–11.8)	5.7 (4-12.3)	5.9 (4.3–15)	0.765^c^
Tryglycerides	133 (61–503)	142 (57–497)	137 (57-1565)	0.982^c^
ALT	15 (10-33)	19 (10–220)	18.5 (8–64)	0.375^c^
AST	20 (6-35)	20.5 (8-205)	19.5 (8-130)	0.607^c^
Urea	30 (15–54)	26 (0.7-168.4)	25 (11–58)	0.436^c^
Creatinine	0.9 (0.5–1.9)	1 (0.48–17.8)	1 (0.5–2.04)	0.499^c^
Uric acid	6.3 (3.1–7.3)	5.85 (2.8–12.10)	5.4 (3.2–13.6)	0.875^c^
VerifyNow PRU	243 (216–272)	134 (97–195)	56.5 (1–93)	**< 0.001** ^**c**^

Data is presented as mean±standard deviation (SD) if normally distributed and median (minimum-maximum) if not normally distributed; *p* < 0.05. ^a^One-way ANOVA; ^c^Kruskal-Wallis

There are two gene polymorphisms examined in this study (Table 3). For the ABCB1 C3435T gene polymorphism, 39.5% were homozygous wildtype (CC), 43.5% were heterozygous (CT), and 16.9% were homozygous variant (TT). There was no significant difference between each genotype and the variability in response to clopidogrel (*p* = 0.858). The allele frequency of C was 61.3%, and the allele frequency of T was 38.7%.

On the other hand, for the ABCB1 C1236T gene polymorphism, 17.8% were homozygous wildtype, 39.5% were heterozygous, and 42.7% were homozygous variant. The allele frequency of C was 37.5%, and the allele frequency of T was 62.5%.

There was no significant association found between ABCB1 genotypes and variability in response to clopidogrel. However, a p-value of < 0.10 was observed for ABCB1 C1236T, prompting further analysis using numerical data from VerifyNow.

**Table 3:** Frequency of ABCB1 Genotypes and Allele with Clopidogrel Response Variability (*n* = 124)

Parameter	*N* (%)	Unresponsive *N* = 16	Responsive *N* = 56	Bleeding risk *N* = 52	*p*
Genotype frequency					
ABCB1 C3435T					0.858^b^
CC	49 (39,5)	6 (37.5)	23 (41.1)	20 (38.5)	
CT	54 (43.5)	8 (50)	25 (44.6)	21 (40.4)	
TT	21 (16.9)	2 (12.5)	8 (14.3)	11 (21.2)	
ABCB1 C1236T					0.070^b^
CC	22 (17.8)	2 (12.5)	5 (8.9)	15 (28.8)	
CT	49 (39.5)	6 (37.5)	27 (48.2)	16 (30.8)	
TT	53 (42.7)	8 (50)	24 (42.9)	21 (40.4)	
Allele frequency					
ABCB1 C3435T					
C	152	0.625	0.634	0.587	
T	(61.3) 96 (38.7)	0.375	0.366	0.413	
ABCB1 C1236T					
C	93 (37.5)	0.313	0.33	0.303	
T	155 (62.5)	0.687	0.67	0.697	

Data presented in n(%) and frequency; *p* < 0.05

^b^Chi-square

A significant association was found between platelet reactivity (as seen on VerifyNow PRU) and ABCB1 C1236T (*p* = 0.031). The lowest median value was observed in the homozygote wildtype group (Table 4). The median values for all groups were < 208 PRU, which does not meet the criteria for clopidogrel resistance. However, in the homozygous wildtype group, a median VerifyNow value of < 95 PRU was found, which corresponds to bleeding risk with clopidogrel administration.

**Table 4:** Association between Platelet Reactivity and ABCB1 C1236T in Clopidogrel Response Variability (*n* = 124)

	ABCB1 C1236T Median (min-max)			*p*
	**CC**	**CT**	**TT**	
Platelet reactivity (VerifyNow PRU)	75.50 (5-237)	124 (7-269)	104 (1-273)	**0.031** ^**c**^

Data presented in median (minimum-maximum); *p* < 0.05. ^c^Kruskal-Wallis

Analysis then continued by grouping ABCB1 C1236 genotype to CC and CT/TT, also response variability to bleeding risk and unresponsive/responsive (Table 5). Study found that subjects with homozygote wildtype (CC) had 3.76 (95% CI 1.408–10.069) higher probability of bleeding risk compared to heterozygote and homozygote variant (CT/TT).

**Table 5:** Association between ABCB1 polymorphism and bleeding risk (*n* = 124)

	Bleeding risk *N* = 52(41.9)	Responsive/unresponsive *N* = 72(58.1)	*p*	OR (95%CI)
ABCB1			**0.006** ^**d**^	**3.76 (1.41–10.07)**
C1236T	15 (28.8)	7 (9.7)		
CC	37 (71.2)	65 (90.3)		
CT/TT				

Data presented in n (%); *p* < 0.05. ^d^Fisher’s-exact; OR: Odds ratio

## Discussion

This study primarily focused on genetic polymorphisms that affects clopidogrel absorption in the intestines, namely ABCB1 with single nucleotide polymorphisms (SNP) C3435T and C1236T. Both SNPs in ABCB1 are known to be most significantly related to clopidogrel response variability based on prior studies. In Indonesia, this study is the first to investigate ABCB1 gene polymorphisms in relation to clopidogrel response variability in ischemic stroke patients.

### Baseline characteristics

The average age of subjects in the three groups ranged from 57.98 to 59.85 (*p* = 0.281), thus comparable across all three groups. In this study, 12.9% of subjects were categorized as non-responders to clopidogrel, which is in line with a previous study that found 15.8% of patients were resistant to clopidogrel [21]. However, this number is lower than previous research on cardiovascular disease populations in Indonesia, which reported a prevalence of clopidogrel resistance of 49.8% [22]. This number is also lower compared to Chinese, Japanese, and Korean populations, with resistance or non-responsiveness to clopidogrel ranging from 20–65% [9]. Meanwhile, 45.2% of subjects in this study were responders to clopidogrel, and 41.9% were at risk of bleeding from clopidogrel administration. Therefore, it can be concluded that the proportion of clopidogrel resistance is lower in Indonesia, with a relatively high proportion of bleeding risk. Additionally, clopidogrel resistance is relatively higher in cardiovascular disease populations compared to stroke populations. This difference could be due to other risk factors for cardiovascular disease and stroke that subsequently affect platelet aggregation, which is used as an indicator of clopidogrel resistance.

In this study, clopidogrel resistance was more prevalent in females (62.5%) compared to males (37.5%), although no significant differences were observed among the three groups. This aligns with previous research that showed the highest proportion of clopidogrel resistance was among females, both in stroke or cardiovascular disease patients [21, 23–25].

In the current study, no significant differences were found among the three groups in terms of demographic and clinical characteristics except for dyslipidemia. Dyslipidemia had a significant difference with clopidogrel response variability (*p* = 0.44), with the highest proportion being in the non-responder group. This is in line with a study by Kang et al., which showed that hyperlipidemia is associated with clopidogrel resistance (*p* = 0.017) and similar studies in coronary artery disease populations [23, 26]. The impact of cholesterol or lipids on platelet activation and biogenesis influences platelet aggregation, which is an indicator of clopidogrel resistance, thus increases the risk of atherosclerosis [26].

In this study, no significant differences were found in other variables such as aspirin consumption history, cilostazol consumption history, stroke history, smoking, type 2 diabetes, hypertension, dyspepsia, body mass index, fatty liver, and uric acid levels. These findings are consistent with previous studies [21]. Regarding the history of aspirin consumption, a study by Legrand et al. also reported that long-term aspirin consumption is not significantly associated with clopidogrel resistance. Similarly, the study found that aspirin resistance is not significantly associated with clopidogrel resistance in multivariate analysis [27].

Based on laboratory tests, hemoglobin levels were significantly associated with clopidogrel response variability. The lowest hemoglobin levels were observed in the non-responder group (*p* < 0.001). This aligns with a study by Legrand et al., which showed that lower hemoglobin levels, particularly < 13.9 g/dL, have a significant association and can be used as a predictor of clopidogrel resistance [27]. Hemoglobin levels can be influenced by hematocrit, where lower hemoglobin is associated with lower hematocrit. It is known that lower hematocrit can affect platelet reactivity testing induced by ADP, especially in samples using whole blood. Both this study and Legrand et al. used VerifyNow PRU with whole blood samples to assess clopidogrel resistance. This could explain the association between lower hemoglobin levels and clopidogrel resistance [28, 29].

In contrast to dyslipidemia, individually total cholesterol, HDL, LDL, and triglycerides did not have a significant association with clopidogrel response variability. This is consistent with our previous research and Su et al. study [21, 30]. However, in coronary artery disease populations, total cholesterol, HDL, and LDL were found to be independent risk factors for clopidogrel resistance [26, 27]. This could be due to the greater influence of increased cholesterol levels on coronary artery disease compared to stroke in both male and female populations [31].

### ABCB1 genotype and allele frequencies

This study found that the most frequent ABCB1 C3435T genotype was heterozygous (CT) at 43.5%, followed by homozygous wild-type (CC) at 39.5%, and homozygous variant (TT) at 16.9%. These results are similar to studies by Budikayanti et al., Istikharah et al., and Syarifah et al. in Indonesian populations with temporal lobe epilepsy and breast cancer [32–34]. Other studies on Asian populations found genotype frequencies for ABCB1 C3435T to be in the ranges of 18-28.86% for homozygous wild-type, 39-53.02% for heterozygous, and 18.12-28% for homozygous variant [35, [36]. The frequency of ABCB1 C3435T alleles in this study was 0.61 for allele C and 0.38 for allele T. Studies on other Asian populations, including China, Malaysia, and India, found allele frequencies in the ranges of 0.38–0.48 for allele C and 0.52–0.62 for allele T [36]. It can be concluded that allele C is more prevalent in the Indonesian population compared to other Asian populations, such as China, Malaysia, and India.

For ABCB1 C1236T, the most frequent genotype in this study was homozygous variant (TT) at 42.7%, followed by heterozygous (CT) at 39.5%, and homozygous wild-type (CC) at 17.8%. This finding is similar to the prevalence in India, where the population is mostly homozygous variant (48.3%), followed by heterozygous (37.9%), and homozygous wild-type (13.8%). However, it differs from other Asian populations, including Turkey, Japan, and Malaysia, where the most frequent genotype was heterozygous (44.6–51%), followed by homozygous variant (29-48.3%), and homozygous wild-type (11–20%). In European populations, such as France and Germany, the most common genotype is heterozygous (49-49.2%), followed by homozygous wild-type (33-34.3%), and homozygous variant (16.4–18%) [37]. The allele frequencies in this study for alleles CT and T were 0.37 and 0.62, respectively, while a study in Turkey showed allele frequencies of 0.45 for allele C and 0.54 for allele T [37]. Therefore, it can be concluded that allele T is more common in the Indonesian population. This is consistent with other Asian populations but differs from European populations.

### ABCB1 genotype and clopidogrel responsiveness

Statistical analysis showed no significant associations for ABCB1 C3435T genotype with clopidogrel responsiveness. This is in line with a meta-analysis study by Su et al., which stated that C3435T polymorphism does not affect platelet reactivity (OR 1.01; 95% CI 0.51–1.97) and Adamiak-Giera et al., which found that C3435T does not affect the levels of inactive clopidogrel metabolites [38, 39]. However, another study by Chen et al. found that the T allele is associated with clopidogrel resistance (*p* = 0.004) [40]. Superscript> Therefore, it can be concluded that the role of ABCB1 C3435T in clopidogrel resistance is inconsistent across studies. In our study, we conducted an analysis of the combination of both C1236T and C3435T genotypes to investigate their potential impact on clopidogrel responsiveness. However, our findings revealed no significant effect on clopidogrel responsiveness associated with this genotype combination. Despite thorough analysis, we did not observe any noteworthy impact on drug response. Consequently, we concluded that the combination of these genotypes does not exert a discernible influence on the response to clopidogrel in our study cohort.

Statistical analysis also showed no significant associations for ABCB1 C1236T genotype with clopidogrel responsiveness. However, as the p-value for ABCB1 C1236T and clopidogrel responsiveness was < 0.10, further analysis was carried out by comparing VerifyNow values with the genotype data. Using the Kruskal-Wallis test, it was found that VerifyNow PRU values were significantly associated with ABCB1 C1236T polymorphism. The lowest median was found in the homozygous wild-type group (*p* = 0.031). The median PRU in this group was < 95 PRU, which is defined as being in the bleeding risk category. Meanwhile, the median VerifyNow PRU in the heterozygous and homozygous variant groups falls within the clopidogrel response category.

Based on these findings, the analysis continued by combining the ABCB1 C1236T polymorphism groups into homozygous wild-type and heterozygous/homozygous variant groups and the clopidogrel response variability groups into bleeding and response/non-response categories. It was found that homozygous wild-type genotypes were significantly associated with bleeding risk from clopidogrel administration (*p* = 0.006). The likelihood of bleeding risk was 3.76 times higher in individuals with homozygous wild-type genotypes compared to those with heterozygous/homozygous variant genotypes (95% CI 1.408–10.068). Previous studies by Mugosa et al., Adamiak-Giera et al., and Cheng et al. did not find an association between ABCB1 C1236T polymorphism and clopidogrel resistance. However, these three studies did not analyze bleeding risk subtypes in clopidogrel [39–41]. The finding of an association between the homozygous wild-type genotype and bleeding risk is a novel finding that should be further investigated in other populations to determine consistency across studies. The limitation of study include few journal discuss about this topic and a bias toward patients with a high risk of bleeding and the absence of clopidogrel plasma level assessment, primarily due to practical constraints such as prohibitive costs and lack of suitable equipment. This limitation restricts our ability to fully understand ABCB1’s impact on clopidogrel responsiveness despite offering valuable insights into genetic factors influencing drug response.

## Conclusion

This study categorized subjects into three groups based on clopidogrel response variability: 12.9% of subjects were non-responders, 45.9% were responders, and 41.9% had a risk of bleeding. The homozygous wild-type genotype at ABCB1 C1236T was associated with a 3.76 times higher risk of bleeding when taking clopidogrel compared to other variants.

Regarding the frequencies of ABCB1 C3435T genotypes, subjects had 35.9% homozygous wild-type, 43.5% heterozygous, and 16.9% homozygous variant genotypes. For C1236T genotypes, the frequencies were 17.8% homozygous wild-type, 39.5% heterozygous, and 42.7% homozygous variant.

### Recommendations

The use of clopidogrel for ischemic stroke cases in Indonesia is beneficial, given the relatively low resistance rates. However, monitoring for bleeding risk is essential, especially considering the relatively higher risk of bleeding compared to other populations. Further research can be conducted using case-control or cohort designs to investigate bleeding events in high-risk groups. Additionally, future studies can assess the role of ABCB1 G2677T/A polymorphisms in clopidogrel response variability. Larger-scale research involving multiple centers is recommended to provide a more comprehensive understanding of the Indonesian population.

## Data Availability

The data generated and analysed during the current study are availabel in grch37 repository. Link available: https://grch37.ensembl.org/Homo_sapiens/Variation/Phenotype?db=core;g=ENSG00000085563;r=7:87133175-87342611;t=ENST00000265724;v=rs1045642;vdb=variation;vf=8271600.

## Abbreviations

ABCB1: ATP-binding cassette subfamily B member-1.
ABC: ATP-binding cassette.
ADP: Adenosine diphosphate.
CYP450: Cytochrome enzymes.
MDR1: Multidrug resistance-1.
P-gp: P-glycoprotein.
SNPs: Single nucleotide polymorphisms.
HPR: High on-treatment platelet reactivity.
